# Data-Driven Clustering of Secretory Bone and Inflammatory Markers to Subphenotype Patients Undergoing Hemodialysis

**DOI:** 10.1016/j.ekir.2026.107019

**Published:** 2026-08-14

**Authors:** Simon Aberger, Ameen Abu-Hanna, Vianda Stel, Nicholas Chesnaye, Emanuel Zitt, Daniel Cejka, Thomas B. Dschietzig, Franz P. Armbruster, Hildegard Hafner-Gießauf, Maximilian Waller, Corinna Schabhüttl, Alexander H. Kirsch, Alexander R. Rosenkranz, Kathrin Eller

**Affiliations:** 1 Division of Nephrology, Department of Internal Medicine, Medical University of Graz, Graz, Austria; 2 Department of Medical Informatics, Amsterdam University Medical Center, University of Amsterdam, Amsterdam, The Netherlands; 3 Department of Methodology, Amsterdam Public Health Research Institute, Amsterdam, The Netherlands; 4 Department of Internal Medicine III, LKH Feldkirch, Feldkirch, Austria; 5 Vorarlberg Institute for Vascular Investigation and Treatment (VIVIT), Feldkirch, Austria; 6 Department of Internal Medicine III, Ordensklinikum Linz Elisabethinen, Linz, Austria; 7 Institute for Physiology and Pathophysiology, Johannes Kepler University Linz, Linz, Austria; 8 Immundiagnostik AG, Bensheim, Germany; 9 Hemodialysis Unit Gießauf, Graz, Austria; 10 Hemodialysis Unit Feldbach, Feldbach, Austria

**Keywords:** Inflammation, hemodialysis, parathyroid hormone, personalized medicine, vascular disease

## Abstract

**Introduction:**

Parathyroid hormone (PTH) remains the primary biomarker used to classify and monitor chronic kidney disease–mineral and bone disorder (CKD-MBD); nonetheless, its reliability for risk stratification and treatment guidance is limited. Multidimensional biomarker clustering that integrates indicators of bone turnover, vascular calcification, inflammatory and oxidative stress may better capture the heterogeneity of clinical phenotypes, improve risk stratification, and inform on new therapeutic approaches in dialysis care.

**Methods:**

We conducted a computational analysis of a multicentric cohort study including 471 patients undergoing hemodialysis. The Partitioning Around Medoids algorithm was used to cluster the cohort by a pathophysiology-based biomarker panel (noxPTH, intact parathyroid hormone [iPTH], osteoprotegerin [OPG], soluble receptor activator of nuclear factor-κB ligand [sRANKL], antioxidative capacity [ImAnOx], oxidative capacity [PerOx], β-Crosslaps [β-CL], and high-sensitivity C-reactive protein [hsCRP]) at baseline. The cluster phenotypes were characterized by anthropometrics, imaging, and clinical data. Cluster-specific outcomes and event patterns were assessed after 1 year.

**Results:**

A total of 4 distinct clusters were identified. Cluster 1 reflected a high bone turnover state with high antioxidative capacity and high bone-specific alkaline phosphatase. Cluster 2 showed low bone turnover with high antioxidative capacity and high sclerostin levels. Cluster 3 exhibited an inflammatory–oxidative phenotype and the highest 1-year mortality; cluster membership improved prediction of events beyond clinical covariates. This phenotype was marked by high aortic calcium burden, vascular disease, markers of vitamin D degradation, low vaccine response, and hepatic steatosis. Cluster 4 showed a balanced biomarker profile and the highest rate of reclassification after 1 year.

**Conclusion:**

Multidimensional biomarker clustering captures heterogeneity in bone and vascular disease phenotypes of CKD-MBD, exhibiting differential mortality risk and event patterns and highlighting inflammation as an important pathway for further studies.

## Graphical abstract

**Figure:**
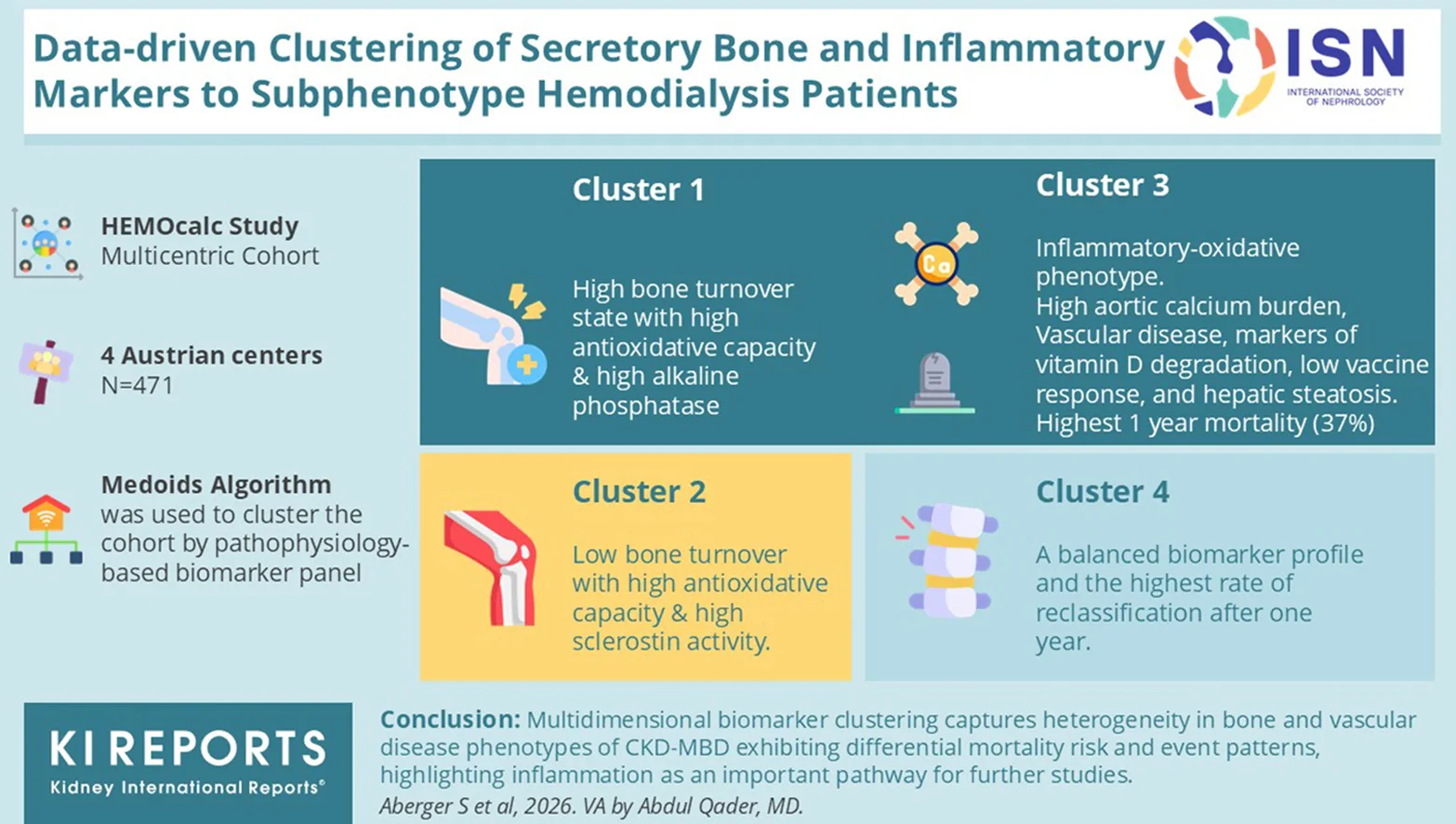

CKD-MBD is a systemic condition in patients undergoing hemodialysis, characterized by dysregulation of bone turnover, mineral metabolism, and vascular calcification, all of which contribute to increased cardiovascular morbidity and mortality.^1^ PTH remains the central biomarker for classifying and monitoring CKD-MBD and for determining secondary hyperparathyroidism and bone metabolic activity.^2^ Longitudinal assessment of markers of bone metabolism has been shown to be associated with outcomes, as shown in the EQUAL study.^3^ However, the clinical use of PTH is limited by analytic variability, accumulation of biologically inactive fragments, post-translational modifications, such as oxidation, and end-organ resistance, which collectively undermine its reliability for risk stratification and as a therapeutic target.^2^

Trials in CKD-MBD frequently involve biochemical outcomes and surrogate clinical outcomes, but hard clinical outcome trials remain scarce.^4^ The EVOLVE study showed that pharmacologic modulation of PTH leads to biochemical improvements, whereas the inference of reductions in mortality or major cardiovascular events was hampered by methodological shortcomings.^5^ Thus, potential beneficial effects of PTH lowering remain unproven. This disconnect between PTH lowering and improved patient outcomes may be attributable to a multifactorial pathophysiology of CKD-MBD. Similarly, the benefit of phosphate control has yet to be proven, as the HiLo study was terminated early because of enrollment issues,^6^ and the PHOSPHATE trial (NCT03573089) is currently ongoing.

Current literature emphasizes the critical roles of inflammation and oxidative stress in CKD-MBD progression. Elevated C-reactive protein (CRP), a marker of systemic inflammation, is independently associated with derangements in bone and mineral metabolism,^7^ as well as increased cardiovascular risk and mortality in patients undergoing hemodialysis.^8^ Oxidative stress further exacerbates vascular and skeletal pathology and may modify PTH structure and function, contributing to PTH resistance and impaired bone response.^2^ Recently, IL-6 inhibition by clazakizumab has been shown to effectively reduce CRP levels in a phase 2b study in patients undergoing hemodialysis,^9^ laying the foundation for a phase 3 trial (NCT05485961).

The need for integrative approaches to advance personalized management has been emphasized in the latest Kidney Disease: Improving Global Outcomes controversies statement on CKD-MBD by Ketteler *et al.*^10^ Given these complexities, we hypothesize that (i) multidimensional biomarker clustering, integrating bone turnover markers, vascular calcification indices, inflammatory mediators, and oxidative stress biomarkers, may advance the pathophysiology-based subclassification of patients undergoing hemodialysis and (ii) could improve prospective risk stratification. We herein report a computational analysis of biomarkers, clinical features, and event patterns, reinforcing the role of pathophysiology-based phenotyping to capture the underlying heterogeneity in a population susceptible to bone disorders and vascular disease.

## Methods

### Study Design

The analysis was conducted as part of the “HEMOcalc” study, a registered, multicentric cohort study (NCT07170761, 34-322ex21/22). Of 475 enrolled patients undergoing maintenance hemodialysis recruited from 4 dialysis centers in Austria (Graz, Linz, Feldbach, Feldkirch), 471 were included in the final analysis. Baseline blood samples were obtained midweek before dialysis and analyzed for a panel of markers related to bone and mineral metabolism, vascular pathology, vitamin D metabolism, oxidative stress, and inflammation. Laboratory analyses, including measurements of noxPTH, iPTH, OPG, RANKL, ImAnOx, PerOx, β-CL, hsCRP, bone-specific alkaline phosphatase (BAP), sclerostin, 1,25(OH)_2_D_3_, 25(OH)D_3_, 24,25(OH)_2_D_3_, and myeloperoxidase, were performed centrally by Immundiagnostik AG Germany (Supplementary Table S1).

Annual clinical follow-up and data collection were conducted through medical record review and linkage with the Austrian Dialysis and Transplant Registry. Uniform electronic case report forms were implemented across all centers using the REDCap data collection platform. In a subset of 148 patients, repeat blood sampling was performed after 1 year with an extended 2-year follow-up period. Additional data at baseline included anthropometric measurements, standardized grading of abdominal aortic calcification (in intervals of 0.5 from grade 0 = without any radiologically visible calcification, grade 1 = calcification of up to 33% of the arterial circumference, grade 2 = calcification of up to 66% of the circumference, to grade 3 = circular calcification)^11^ by noncontrast computed tomography imaging of the abdominopelvic region, and presence of valvular calcification on echocardiography.

The primary outcome was all-cause mortality after 1 year. Secondary outcomes included cause-specific death, major adverse cardiovascular events (MACE) (4-point MACE), incident fractures, hospitalization for infection or heart failure, and atherosclerotic cardiovascular disease interventions.

### Cluster Analysis

The clustering analysis aimed to identify patient subgroups representing pathophysiologic pathways of vascular and bone disease, on the basis of 7 prespecified biomarkers—noxPTH, β-CL, OPG, sRANKL, hsCRP, lmAnOx, and PerOx. Variable selection followed the pathophysiology-based clustering strategy described by Wagner *et al.*^12^ and was designed to capture complementary biological domains, including bone turnover, osteoblast/osteoclast signaling, inflammation, oxidative stress, and antioxidative capacity. To maintain a parsimonious model appropriate for the cohort size, biomarkers providing overlapping information (Spearman correlation coefficient > 0.5), including iPTH, BAP, sclerostin, vitamin D metabolites, and myeloperoxidase, were excluded from the clustering algorithm to reduce redundancy and collinearity but were analyzed descriptively across the identified clusters. Before distance computation, variables were Winsorized at the 1^st^ and 99^th^ percentiles to reduce the influence of extreme outliers and then center-level standardized (mean 0; SD 1). The range-normalized Manhattan distances were calculated, and clustering was performed using Partitioning Around Medoids through the R package *cluster,* a robust alternative to k-means clustering.^13^ The optimal number of clusters was determined on the basis of silhouette width analysis. Cluster stability was evaluated using bootstrap resampling^14^ with 1000 replicates. *Post hoc* visualization of cluster profiles was performed using radar charts showing *z*-scores. Differences in clustering variables, biomarkers, metabolites, and clinical characteristics were explored descriptively across the identified clusters. Sensitivity analyses included leave-one-center-out analysis and longitudinal validation of the clustering results in a subset of patients with repeated measurements of cluster markers after 1 year.

### Statistical Analysis

Baseline characteristics were summarized using descriptive statistics—mean ± SD or median with interquartile range for continuous variables and frequencies with percentages for categorical variables. Differences among clusters were assessed using the Kruskal–Wallis test or one-way ANOVA, as appropriate, for continuous variables and the χ^2^ test for categorical variables. *P* values were adjusted for multiple testing using the Benjamini–Hochberg false discovery rate procedure.

Cumulative survival differences between clusters were assessed through Kaplan-Meier analysis, censoring for kidney transplantation. Secondary outcomes were analyzed using the cumulative incidence function, accounting for competing events (cause-specific death and kidney transplantation).

For deeper analysis of the prognostic potential of cluster membership for all-cause mortality, we used Fine-Gray subdistribution hazards models accounting for kidney transplantation as a competing risk. An incremental modeling strategy was used to examine whether cluster membership captures a similar proportion of prognostic information as the individual cluster components when added to baseline clinical variables. Backward variable selection for the baseline model was guided by the Akaike Information Criterion. Model 1 included Akaike Information Criterion–selected baseline variables—age, body mass index, dialysis vintage, heart failure status, chronic obstructive pulmonary disease status, and serum albumin. Model 2 added the individual cluster components. Cluster membership (with a value of 1 for patients belonging to the respective cluster and otherwise 0) was then added to the baseline variables (Model 3) and individual cluster components (Model 4). Missing baseline variables (<10% missingness) were imputed using the multiple imputation by chained equations algorithm.

A two-tailed *P* value < 0.05 was accepted as statistically significant. Statistical analyses were performed using the R statistical language (version 4.5.2).

## Results

### Unsupervised Clustering Revealed 4 Different Clusters in the Hemodialysis Cohort

A total of 471 patients undergoing hemodialysis from 4 Austrian centers were included in the final analysis (Supplementary Figure S1); baseline characteristics are shown in Supplementary Table S2. Using unsupervised clustering of 7 prespecified, pathophysiology-based biomarkers reflecting bone turnover, oxidative stress, and inflammation (noxPTH, β-Crosslaps, OPG, sRANKL, hsCRP, ImAnOx, PerOx), we identified 4 biologically distinct patient clusters (Jaccard similarity indices 0.73–0.88; mean, 0.80). Cluster profiles showed distinct patterns across bone turnover, inflammation, and oxidative stress markers (Figure 1). Cluster 1 had the highest noxPTH and β-CL levels, suggesting a high-bone-turnover state, whereas Cluster 2 exhibited a low bone turnover profile with preserved antioxidant capacity. Cluster 3 was characterized by a low bone turnover profile with the highest hsCRP and PerOx, and Cluster 4 showed intermediate marker levels (Figure 1). The iPTH–noxPTH correlation was strong across all clusters (Supplementary Figure S2). Patients were well balanced across centers within each cluster (Supplementary Table S3). Leave-one-center-out analysis validated the final clustering solution to be both stable across centers and generalizable to unseen data, as evidenced by consistently high adjusted rand index values (Supplementary Table S4).

**Figure 1:**
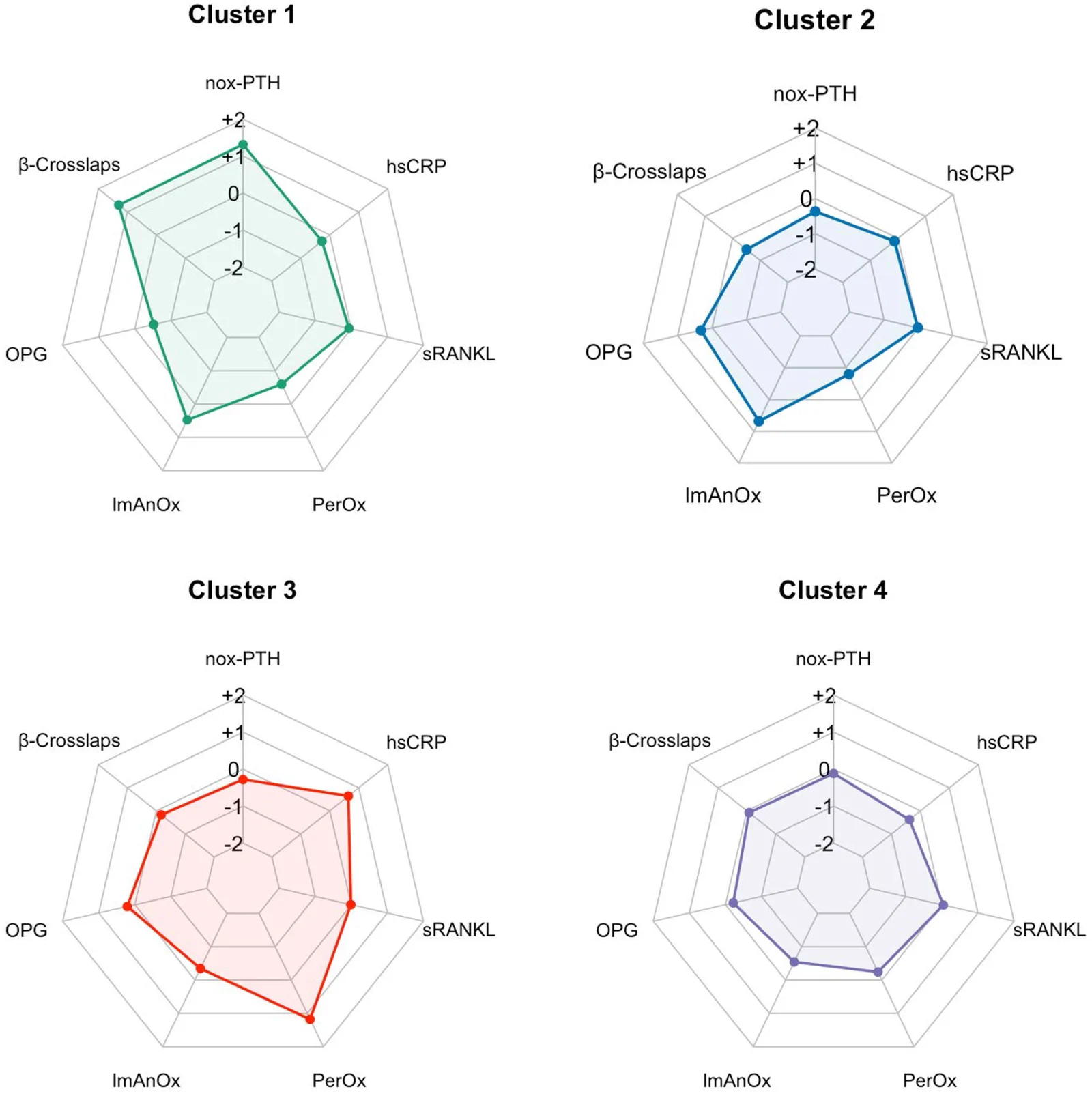
Distribution of feature variables across the 4 clusters. Radar plots display the mean standardized value (*z*-score) of each marker for each cluster. A *z*-score of 0 represents the overall study mean; positive values indicate higher-than-average levels and negative values indicate lower-than-average levels. The overall shape of each plot highlights the relative biomarker profile of each cluster and facilitates comparison of marker patterns across clusters. ImAnOx, antioxidative capacity; hsCRP, high-sensitivity C-reactive protein; OPG, osteoprotegerin; PerOx, oxidative capacity; PTH, parathyroid hormone; sRANKL, receptor activator of nuclear factor-κB ligand.

### Cluster Characteristics Including Cardiovascular Morbidity, Infections, and Overall Mortality

The assigned clusters were then used to compare anthropometric data, clinical characteristics, calcification burden, and additional biomarkers to reach a deeper understanding of the underlying phenotypes (Table 1). Cluster 1 was younger (median age, 60 years), had lower dialysis vintage, lower cardiovascular comorbidities, and accordingly a lower aortic calcification grading (Figure 2a). Cluster 1 also reached the highest BAP levels with high serum phosphate and low sclerostin levels. Patients in Cluster 2 and Cluster 3 were older (median age, 73 and 72 years, respectively) with higher comorbidity burden, including coronary artery and peripheral artery disease. Cluster 2 and Cluster 3 showed a low bone turnover profile, but sclerostin was significantly elevated only in Cluster 2. Cluster 3 had the highest aortic calcification grading (Figure 2a) and valvular calcification burden (Table 1). In addition, Cluster 3 showed the lowest serum albumin (Figure 2b), highest proportion of hepatic steatosis, and the lowest anti-HBs (antibodies against hepatitis B surface antigen) titers (Table 1). Furthermore, a higher number of central venous catheter vascular accesses was notable (Table 1). Cluster 4 had a median age of 70 years with a lower dialysis vintage similar to Cluster 1 and an otherwise intermediate marker and feature profile. Serum calcium, hemoglobin levels, and markers of iron repletion were balanced between groups, with equal rates of erythropoietin-stimulating agents and intravenous iron prescription. Concerning medication differences, the proportion of patients receiving calcimimetics (50.7%) and phosphate binders (84%) was highest in Cluster 1 (Table 1). In Clusters 2, 3, and 4, calcimimetics were used in 27%–34% of patients, whereas phosphate binders and active vitamin D were prescribed in about 60% of patients (Table 1). The use of antiresorptive treatment (denosumab, bisphosphonates) was overall low without significant differences between clusters, ranging from 1.3% in Cluster 1 to 3.4% in Cluster 2 (Table 1).

**Table 1:** Baseline characteristics of the cohort stratified into 4 clusters

Biological parameters	Cluster 1 (*n* = 75)	Cluster 2 (*n* = 117)	Cluster 3 (*n* = 138)	Cluster 4 (*n* = 141)	*P* value (adjusted)
Age (y)	61.0 (45.0–71.0)	73.0 (64.0–80.0)	72.0 (64.0–78.0)	70.0 (63.0–77.0)	< 0.01 (< 0.01)
BMI	25.3 (22.3–29.9)	25.1 (21.9–28.4)	24.9 (21.4–29.4)	25.5 (22.8–28.9)	0.64 (0.99)
Male sex (%)	52 (69.3%)	79 (67.5%)	82 (59.4%)	95 (68.3%)	0.32 (0.99)


Dialysis parameters	Cluster 1 (n = 75)	Cluster 2 (n = 117)	Cluster 3 (n = 138)	Cluster 4 (n = 141)	P value (adjusted)
Dialysis vintage [mo]	25.7 (8.9–46.5)	32.1 (12.2–74.2)	31.8 (15.6–64.8)	26.2 (10.3–42.7)	< 0.01 (0.01)
HD [h/wk]	11.7 (1.1)	11.6 (1.1)	11.5 (1.3)	11.7 (0.9)	0.28 (0.99)
Blood flow [ml/min]	275.6 (31.8)	272.6 (34.5)	277.5 (33.4)	272.2 (41.6)	0.49 (0.98)
RKF (ml/d)	633.3 (529.7)	591.8 (570.5)	644.8 (509.2)	742.3 (572.0)	0.12 (0.96)
ktV (single pool)	1.3 (0.3)	1.3 (0.3)	1.3 (0.3)	1.3 (0.3)	0.47 (0.99)
AV fistula (%)	49 (65.3%)	63 (54.3%)	59 (44.4%)	78 (56.5%)	0.01 (0.22)


CVD burden	Cluster 1 (*n* = 75)	Cluster 2 (*n* = 117)	Cluster 3 (*n* = 138)	Cluster 4 (*n* = 141)	*P* value (adjusted)
Smoking status (%)	12 (21.8%)	7 (8.9%)	11 (11.7%)	16 (15.1%)	0.04 (0.58)
CHF (%)	11 (14.9%)	29 (24.8%)	44 (31.9%)	31 (22.1%)	0.04 (0.64)
COPD (%)	5 (6.7%)	8 (6.9%)	21 (15.2%)	22 (15.6%)	0.10 (0.99)
CAD (%)	22 (29.3%)	42 (37.9%)	65 (47.1%)	51 (36.2%)	0.04 (0.06)
PAD (%)	14 (18.7%)	42 (35.9%)	47 (34.0%)	42 (29.8%)	< 0.01 (0.03)
Carotid disease (%)	12 (16.0%)	30 (25.6%)	25 (18.1%)	33 (33%)	0.25 (0.31)
AHT (%)	67 (90%)	116 (99.1%)	132 (95.7%)	129 (91.5%)	0.04 (0.61)
Diabetes (%)	22 (29.3%)	60 (51.3%)	61 (44.2%)	58 (41.1%)	0.02 (0.04)
Hepatic steatosis (%)	11 (14.7%)	13 (11.3%)	28 (20.2%)	16 (11.3%)	< 0.01 (0.05)
Aortic calcification grade	1.6 (1.0)	1.7 (1.0)	2.2 (0.8)	1.8 (0.9)	0.07 (0.09)
Valve calcification (%)	7 (19.4%)	26 (41.9%)	34 (55.0%)	20 (33.3%)	< 0.01 (0.04)

Medication	Cluster 1 (*n* = 75)	Cluster 2 (*n* = 117)	Cluster 3 (*n* = 138)	Cluster 4 (*n* = 141)	*P* value (adjusted)
Calcimimetics (%)	38 (50.7%)	32 (27.4%)	43 (31.2%)	48 (34.0%)	0.01 (0.08)
Active VD (%)	40 (53.3%)	73 (62.4%)	79 (57.2%)	84 (59.6%)	0.02 (0.07)
Phosphate binders (%)	63 (84.0%)	91 (77.7%)	92 (66.7%)	100 (70.9%)	0.87 (0.99)
Antiresorptives (%)	1 (1.3%)	4 (3.4%)	4 (2.9%)	3 (2.1%)	0.14 (0.58)
ESA (%)	47 (63%)	81 (69%)	96 (69%)	91 (65%)	0.26 (0.67)
IV iron (%)	37 (49%)	59 (50%)	63 (46%)	63 (45%)	0.31 (0.74)

Laboratory parameters	Cluster 1 (*n* = 75)	Cluster 2 (*n* = 117)	Cluster 3 (*n* = 138)	Cluster 4 (*n* = 141)	*P* value (adjusted)
iPTH (pg/ml)	594.5 (441.9–744.1)	198.7 (135.9–271.9)	213.0 (111.7–375.1)	286.4 (194.9–408.9)	< 0.01 (< 0.01)
noxPTH (pg/ml)	388.3 (279.5–503.2)	127.9 (81.9–183.8)	127.2 (68.4–239.5)	188.9 (120.7–260.9)	< 0.01 (< 0.01)
iPTH:noxPTH ratio	1.5 (0.28)	1.6 (0.24)	1.6 (0.33)	1.5 (0.28)	0.32 (0.99)
Beta-CL (ng/ml)	3.2 (2.4–3.8)	1.2 (0.8–1.6)	1.5 (1.1–2.1)	1.6 (1.2–2.1)	< 0.01 (< 0.01)
OPG (pmol/ml)	15.0 (11.4–24.9)	28.0 (21.5–35.9)	24.9 (19.9–36.2)	19.9 (14.2–27.5)	< 0.01 (< 0.01)
lmAnOx (μmol/ml)	334.4 (305.2–351.6)	351.0 (322.1–369.8)	287.4 (268.8–321.2)	289.3 (274.7–308.7)	< 0.01 (< 0.01)
PerOx (μmol/ml)	184.8 (65.6–380.6)	129.5 (50.0–226.5)	557.45 (471.5–626.7)	282.9 (210.5–351.4)	< 0.01 (< 0.01)
sRANKL (ng/ml)	12.9 (5.7–71.7)	13.5 (4.5–115.3)	17.9 (6.1–87.7)	14.9 (5.5–106.5)	< 0.01 (0.03)
hsCRP (mg/l)	4.0 (1.3–9.1)	5.0 (2.0–10.0)	15.5 (7.0–25.9)	4.0 (2.0–6.0)	< 0.01 (< 0.01)
MPO (IU/ml)	300.5 (237.6–364.9)	282.6 (210.4–382.0)	300.2 (221.8–384.7)	293.1 (220.5–356.6)	0.56 (0.99)
1,25-D3 (ng/ml)	11.0 (6.6–17.8)	10.3 (5.6–19.4)	13.3 (7.1–21.1)	11 (6.4–17.6)	0.41 (0.99)
24,25-D3 (ng/ml)	0.3 (0.2–0.5)	0.4 (0.2–0.5)	0.4 (0.2–0.6)	0.3 (0.2–0.5)	< 0.01 (0.10)
25-D3 (ng/ml)	28.0 (13.1–46.3)	31.1 (14.8–43.3)	27 (13.3–43.5)	28.1 (14.7–43.2)	0.01 (0.26)
VMR (25/24.25D3)	82.5 (41.4)	76.7 (45.5)	60.0 (43.9)	68.4 (41.3)	< 0.01 (< 0.01)
BAP (ng/ml)	26.3 (19.6–39.8)	16.6 (12.5–27.1)	20.2 (14.8–26.6)	18.0 (13.3–26.5)	< 0.01 (< 0.01)
Sclerostin (pmol/l)	89.1 (85.9–115.0)	136.7 (104.2–189.1)	100.6 (69.6–137.7)	106.2 (79.3–146.1)	< 0.01 (< 0.01)
Phosphate (mmol/l)	2.1 (1.7–2.4)	1.7 (1.5–2.1)	1.7 (1.4–2.1)	1.7 (1.5–2.1)	<0.01 (<0.01)
Calcium (mmol/l)	2.09 (2.0–2.4)	2.10 (2.0–2.4)	2.10 (2.0–2.4)	2.10 (2.0–2.4)	0.98 (0.99)
Albumin (g/dl)	4.2 (3.9–4.4)	4.1 (3.7–4.3)	3.7 (3.4–3.9)	4.0 (3.7–4.3)	< 0.01 (< 0.01)
ALP (U/L)	105.0 (73.0–133.0)	81.5 (63.1–108.2)	95.0 (78.5–120.1)	81.0 (64.2–103.4)	< 0.01 (< 0.01)
Hemoglobin (g/dl)	11.4 (10.6–12.0)	11.7 (10.9–12.4)	10.9 (10.1–11.8)	10.9 (10.4–11.8)	< 0.01 (< 0.01)
Ferritin (μg/l)	484.0 (263.0–738.5)	514.0 (282.0–803.8)	524.5 (318.0–837.0)	507.5 (303.5–767.0)	0.52 (0.99)
TSAT (%)	23.2 (17.9–32.0)	24.0 (18.9–32.1)	21.5 (16.0–27.1)	24.0 (17.1–31.0)	0.03 (0.81)
Cholesterol (mg/dl)	156.0 (133.5–193.0)	144.0 (120.5–182.8)	146.5 (119.2–183.3)	149.0 (121.5–182.0)	0.24 (0.99)
LDL (mg/dl)	87.0 (62.8–107.8)	75.0 (55.0–100.1)	72.5 (54.9–106.5)	80.0 (51.3–107.3)	0.09 (0.99)
HDL (mg/dl)	42.1 (34.5–53.9)	43.3 (34.9–51.5)	42.0 (36.2–47.3)	41.5 (34.3–49.0)	0.68 (0.99)
Anti-HBs (IU/ml)	78.0 (2.0–167.5)	31.0 (2.0–112.5)	18.5 (2.0–153.8)	68.0 (2.0–314.5)	0.01 (0.09)

AHT, arterial hypertension; ALP, alkaline phosphatase; Anti-HBs, antibodies against hepatitis B surface antigen; AV fistula, arteriovenous fistula; BAP, bone-specific alkaline phosphatase; beta-CL, β-Crosslaps; BMI, body mass index; CAD, coronary artery disease; CHF, chronic heart failure; COPD, chronic obstructive pulmonary disease; CVD, cardiovascular disease; hsCRP, high-sensitivity C-reactive protein; ESA, erythropoiesis-stimulating agent; HD, hemodialysis; HDL, high-density lipoprotein; iPTH, intact parathyroid hormone; IV iron, intravenous iron; LDL, low-density lipoprotein; lmAnOx, lipid-modulated antioxidative capacity; MPO, myeloperoxidase; noxPTH, non-oxidized parathyroid hormone; OPG, osteoprotegerin; PAD, peripheral arterial disease; RKF, residual kidney function; sRANKL, soluble receptor activator of nuclear factor-κB ligand; TSAT, transferrin saturation; VMR, vitamin D metabolite ratio (25-hydroxyvitamin D/24,25-dihydroxyvitamin D).

Values are presented as median (Q1–Q3) for age, dialysis vintage, BMI, and all laboratory parameters and as mean (SD) for hemodialysis hours/week, blood flow, RKF, Kt/V, PTH/noxPTH ratio, VMR, and abdominal aortic calcification grade (0–3). The VMR (25(OH)D_3_ / 24,25(OH)_2_D_3_) reflects CYP24A1-mediated catabolism, where a lower ratio indicates proportionally greater conversion to the inactive 24,25-dihydroxyvitamin D metabolite. *P* values were calculated using the Kruskal–Wallis test or one-way ANOVA, as appropriate. Benjamini–Hochberg false discovery rate–adjusted *P* values are presented in parentheses.

**Figure 2:**
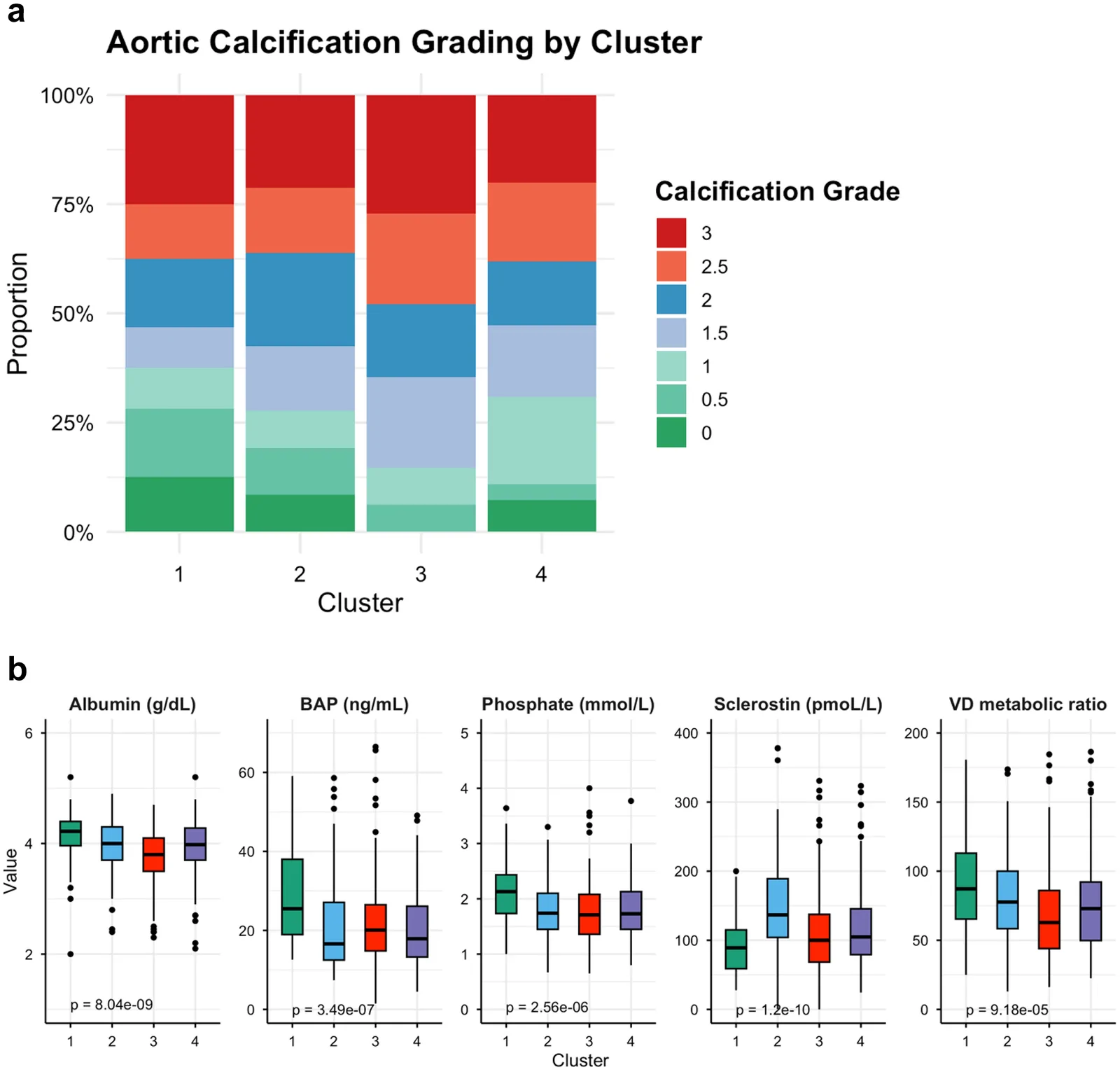
Comparison of abdominal aortic calcium scores and selected variables across clusters. (a) Abdominal aortic calcium scores presented as a bar chart. Kruskal–Wallis with Dunn *post hoc* test showed a significant difference between Cluster 1 and Cluster 3 (*P* = 0.00092). (b) Boxplots showing the distribution of additional variables of interest across the 4 clusters. The *P* values were calculated using the Kruskal–Wallis test. The VMR was calculated as 25-D_3_ / 24,25-D_3_. BAP, bone-specific alkaline phosphatase; VD, vascular disease.

### Patients Undergoing Hemodialysis Grouped in Cluster 3 Exhibit the Highest 1-Year Mortality

The Kaplan-Meier analysis showed significant survival differences by cluster (log-rank *P* < 0.001) (Figure 3), with Cluster 3 exhibiting the highest 1-year mortality of 37.1 % (Supplementary Table S5). Fine–Gray competing risk regression showed that inclusion of Cluster 3 membership in the baseline clinical model was associated with a markedly increased risk of 1-year mortality (hazard ratio, 3.01; 95% confidence interval, 1.88–4.65; *P* < 0.01) and improvement in model discrimination (area under the curve, 0.84; integrated discrimination improvement = 0.06; *P* = 0.04 vs baseline) (Supplementary Table S6). When including cluster components and cluster membership, Cluster 3 remained a meaningful predictor of increased 1-year mortality (hazard ratio, 1.97; 95% confidence interval, 0.93–3.58; *P* = 0.08) (Table 2) without further improvement in model performance (integrated discrimination improvement = 0.01) (Supplementary Table S6). Cumulative incidence analysis with competing risks showed that Cluster 3 had the highest rates of cardiovascular and sepsis-related death (13.2% and 5.8% at 1 year, respectively), as well as MACE (16.3%) and infection-related hospitalizations (24.6%) (Table 3). No significant cluster differences were observed in atherosclerotic cardiovascular disease interventions, fracture-related hospitalizations, or heart failure admissions. A *post hoc* conceptual overview integrating the biological interpretation of each cluster with suggested vascular mechanisms, event patterns, and exploratory therapeutic targets across the 4 phenotypes is provided in Figure 4.

**Figure 3:**
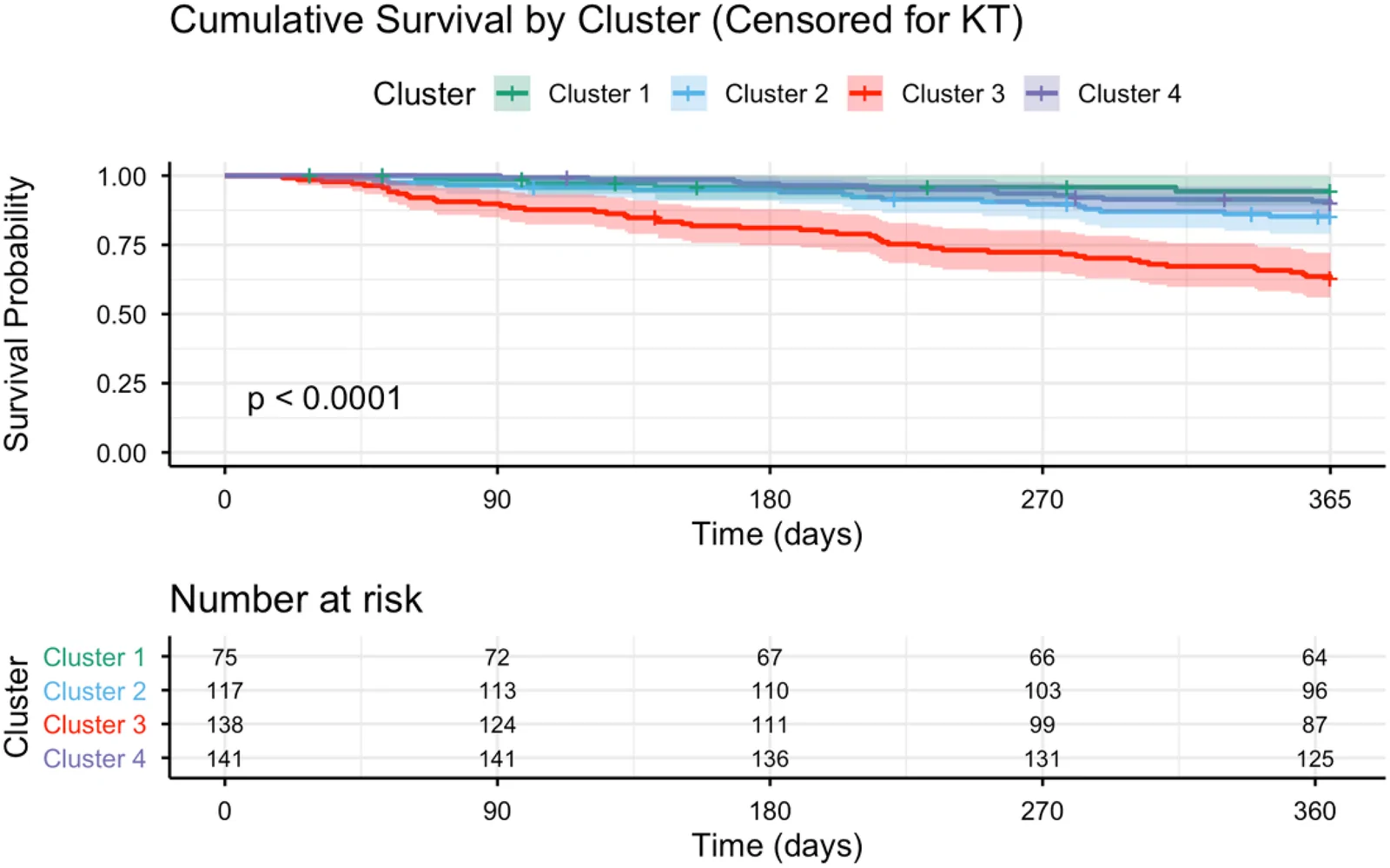
Cluster-specific differences in all-cause mortality. Kaplan–Meier survival curves stratified by cluster, showing cumulative survival with log-rank *P* value. Patients receiving kidney transplantation (*n* = 16) were censored at the time of transplantation. The median follow-up time was 365 days. KT, kidney transplantation.

**Table 2:** Fine–Gray competing-risk analysis of Cluster 3 membership added to baseline variables and individual cluster components

Model	Covariates	Variable	HR	95% CI	*P* value	AIC	AUC
Model 1	Baseline	NA	-	-	-	994	0.79
Model 2	Model 1 + Cluster components	NA	-	-	-	979	0.83
Model 3	Model 1 + Cluster 3	Cluster 3	3.01	1.88–4.65	<0.01	972	0.84
Model 4	Model 2 + Cluster 3	Cluster 3	1.97	0.93–3.58	0.08	975	0.81

AIC, Akaike Information Criterion; AUC, area under the curve; CI, confidence interval; HR, hazard ratio.

HRs with 95% CI for death within 1 year, AIC, and AUC from incremental Fine–Gray competing-risk models (Models 1–4) are shown. Model 1 includes age, dialysis vintage, bidy mass index, congestive heart failure, chronic obstructvie pulmonary disease, and serum albumin. Model 2 includes Model 1 plus noxPTH, β-Crosslaps, OPG, ImAnOx, PerOx, sRANKL, and hsCRP. Models 3 and 4 further include Custer 3 as a dummy variable. Lower AIC indicates better model fit. Higher AUC indicates better model discrimination.

**Table 3:** Cumulative incidence of secondary outcomes across clusters

Outcome	Cluster 1	Cluster 2	Cluster 3	Cluster 4	*P* value
CV death	1/75 (1.3%)	11/117 (7.8%)	19/138 (13.2%)	6/141 (4.3%)	< 0.01
Sepsis death	1/75 (1.3%)	1/117 (0.9%)	7/138 (5.8%)	0/141 (0.0%)	0.01
Other death	2/75 (2.8%)	4/117 (3.4%)	18/138 (11.9%)	7/141 (3.6%)	< 0.01
MACE	5/75 (7.1%)	14/117 (8.9%)	26/138 (16.3%)	12/141 (8.6%)	0.03
Lower limb event	5/75 (4.0%)	11/117 (8.7%)	12/138 (4.8%)	9/141 (4.3%)	0.09
ASCVD intervention	8/75 (6.7%)	15/117 (11.1%)	16/138 (8.7%)	22/141 (12.8%)	0.67
HF hospitalization	2/75 (2.7%)	3/117 (2.6%)	11/138 (5.8%)	5/141 (3.5%)	0.13
Infection hospitalization	10/75 (13.3%)	27/117 (20.5%)	40/138 (24.6%)	30/141 (17.0%)	0.07
Fractures	3/75 (2.7%)	7/117 (5.8%)	7/138 (3.4%)	8/141 (2.1%)	0.09

ASCVD, atherosclerotic cardiovascular disease; CV, cardiovascular; HF, heart failure; MACE, major adverse cardiovascular events.

1*-*year cumulative incidence for first-time cardiovascular death, sepsis death, other death, major adverse cardiovascular events (4-point MACE), atherosclerotic cardiovascular disease interventions, hospitalizations for heart failure or infection, and fractures are presented as observed events per cluster (*n*/*N*) with cumulative incidence estimates (%) in parentheses. Cause-specific deaths and kidney transplantation were treated as competing events; therefore, cumulative incidence estimates may differ from crude event proportions. P values were derived from the Gray test.

**Figure 4:**
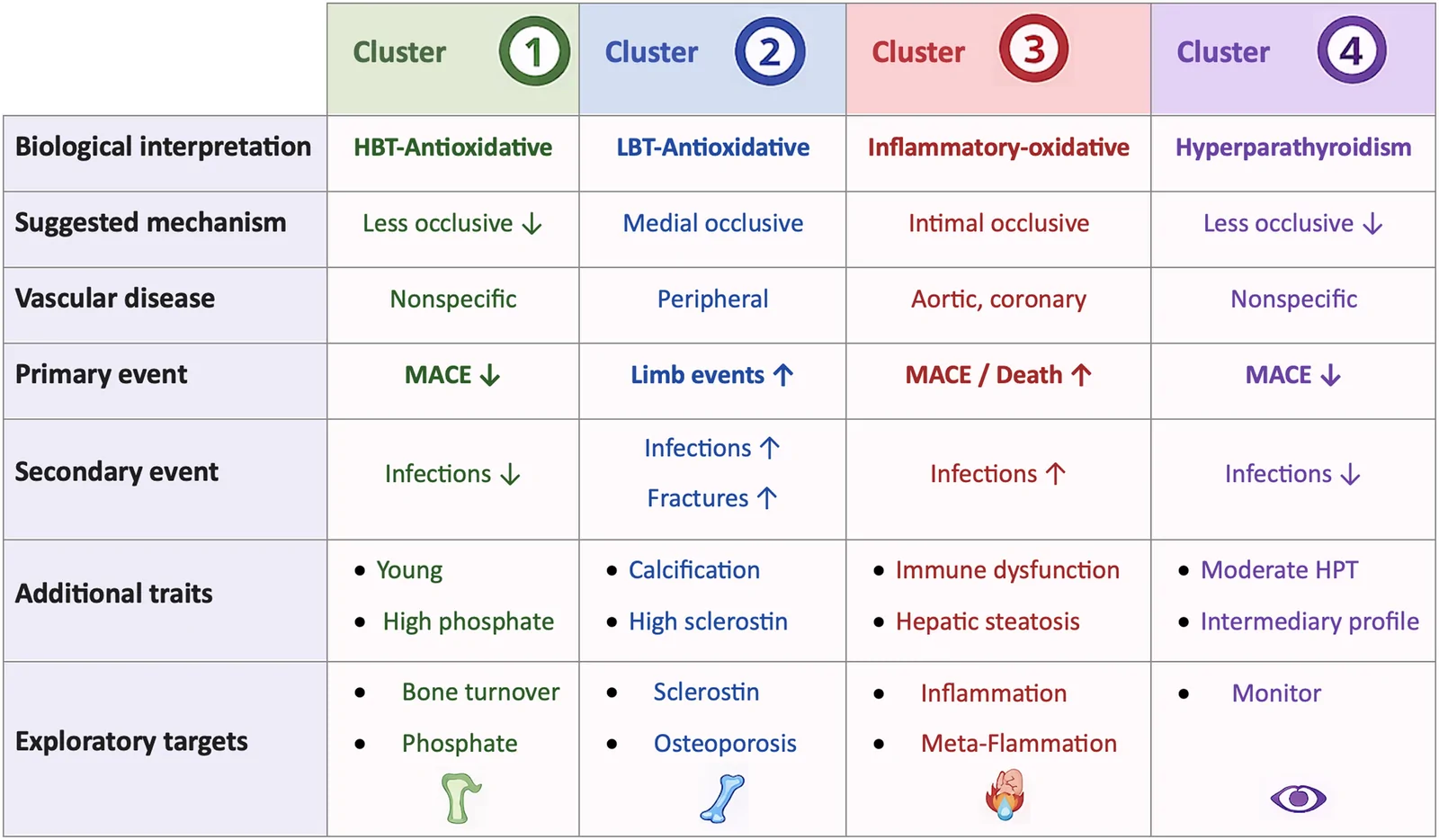
Hypothesis-generating conceptual framework of biomarker-derived cluster phenotypes along the bone–vascular disease axis. Four clusters were identified according to dominant pathophysiologic pathways related to bone and vascular disease. Each cluster was subsequently characterized by suggested vascular mechanism, major clinical events, and characteristic patient traits. On the basis of *post hoc*, data-informed interpretations of the prevailing biological mechanisms within each cluster, exploratory therapeutic targets were proposed to illustrate how pathophysiology-based phenotyping could generate hypotheses for individualized management strategies. HBT, high bone turnover; LBT, low bone turnover; MACE, major adverse cardiovascular events.

### Validation of the Clustering Approach by Remeasuring Variables in Patients Undergoing Hemodialysis After 1 Year

For internal validation, clustering variables were remeasured in a subset of 148 patients at year 1 to repeat clustering, along with assessment of all-cause mortality during and an extended observation period for another year. Clustering at year 1 confirmed the cluster structure (Supplementary Figure S3). Compared with baseline, around 40% of patients in Clusters 1 and 2 changed their cluster status after 1 year, predominantly into Cluster 4 (Supplementary Table S7). The highest reclassification rate was seen in Cluster 4 (68%). Cluster 3 was more stable, with only 32% of patients changing their cluster status (Supplementary Figure S4). Assessment of year-2 mortality confirmed the significantly higher mortality in patients assigned to Cluster 3 at year 1 (Figure 5).

**Figure 5:**
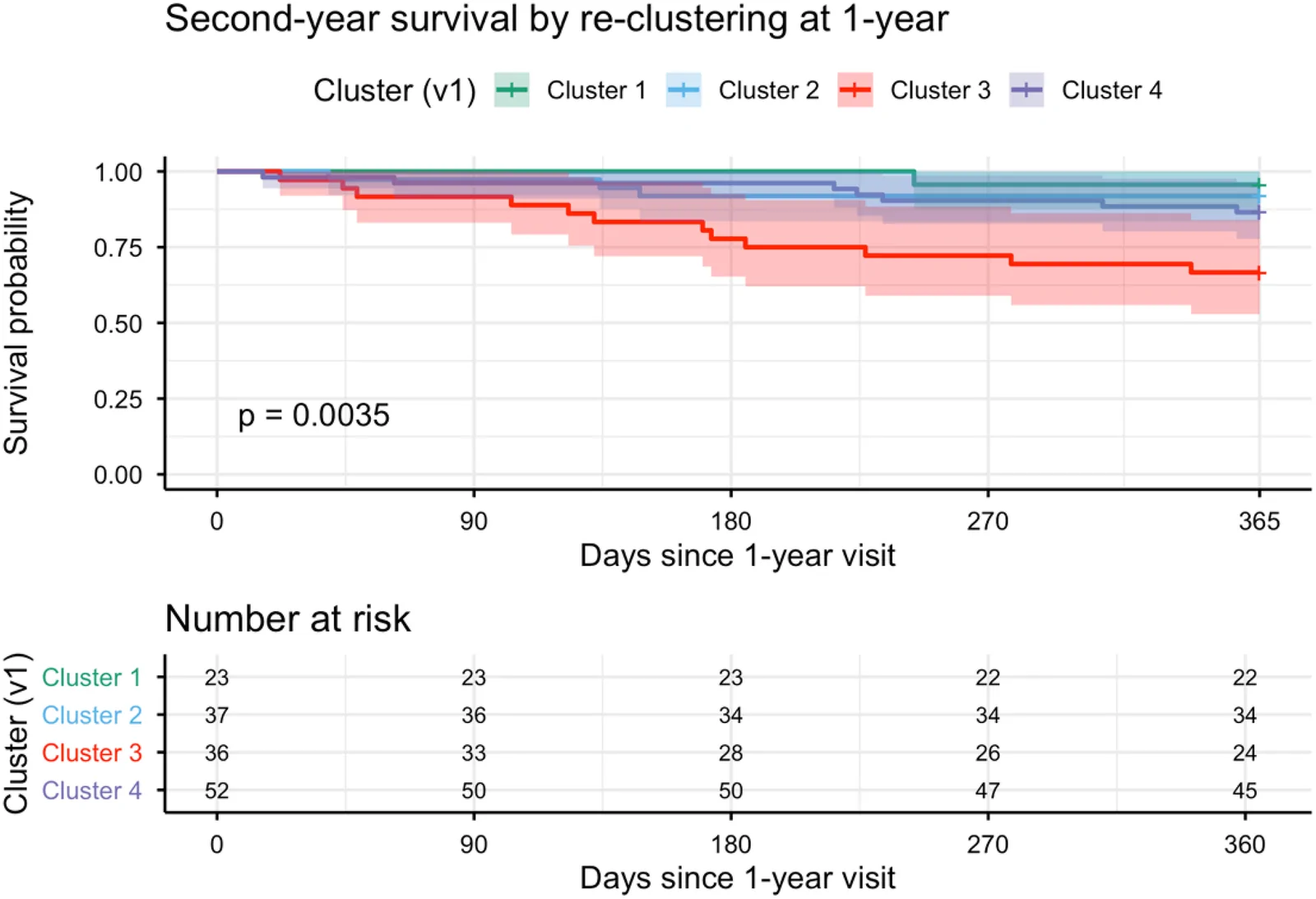
Reproducibility of cluster-specific all-cause mortality after reclustering at 1 year. Kaplan–Meier survival curves showing cumulative survival during extended follow-up in patients restratified into clusters after the first year, with log-rank *P* value. Patients receiving kidney transplantation (*n* = 1) were censored at the time of transplantation.

## Discussion

This multicenter cohort comprises 471 prevalent patients undergoing hemodialysis with detailed documentation of clinical characteristics, comorbidity burden, and treatment regimens in accordance with current guidelines. By integrating multidimensional biomarker data reflecting bone metabolism, oxidative stress, and systemic inflammation, we identified 4 reproducible and biologically coherent patient clusters. These clusters diverged in both pathophysiologic profiles and clinical trajectories, supporting the relevance of pathophysiology-based subclassification in patients undergoing hemodialysis to reflect heterogeneity and outcomes in bone and vascular diseases. Systemic inflammation stands out as a potential risk feature to be addressed in future studies.

Cluster 1 displayed high bone turnover with preserved antioxidant status and low vascular risk. This aligns with evidence showing that high PTH and turnover markers often characterize younger patients with lower calcification burden.^15^ Cluster 1 is demographically and clinically similar to the populations enrolled in the EVOLVE trial (cinacalcet) and the phase 3 trials of etelcalcetide,^16^ in which relatively low event rates occurred. Concordantly, Cluster 1 in our cohort exhibited lower mortality, fewer MACE events, and less aortic calcification. The high anti-HBs titers may indicate preserved immune competence and less immune exhaustion.^17^

Cluster 2 exhibited an adynamic bone phenotype, consistent with older age, lower bone turnover markers, and greater comorbidity burden. Although low bone turnover is linked to fracture risk and multimorbidity in CKD,^18^ mortality in this cluster remained low and comparable with Clusters 1 and 4, despite the highest age, longest dialysis vintage, and marked calcification burden. The high vascular intervention rate of 11.1% was substantially driven by lower limb events (8.9%), which may be consistent with a predominance of Mönckeberg-type medial arterial calcification, a process driven by vascular smooth muscle cell transdifferentiation, characteristically independent of systemic inflammation.^19^ The absence of elevated hsCRP with preserved antioxidative capacity, low bone turnover, and elevated sclerostin is a plausible biological features in line with this calcification pattern. Given the low bone turnover and high 1-year fracture rate of 5.8%, the elevated sclerostin levels may be relevant, as romosozumab (a monoclonal antibody targeting sclerostin) has shown promise in improving bone turnover and bone mineral density in patients undergoing hemodialysis.^20^ Cluster 2 may represent an interesting target population for future studies examining fracture- and bone structure-related outcomes in a relatively low cardiovascular risk setting.

Cluster 3 showed a pronounced inflammatory–oxidative stress signal and high cardiovascular event rates. This subgroup is consistent with literature connecting oxidative stress and osteo-immunologic pathways (i.e., OPG–RANKL axis) with progressive vascular calcification, intimal artery disease, and mortality in dialysis,^21^^,^^22^ thus resembling a CKD-associated cardiovascular disease phenotype.^10^ The marked elevation of hsCRP and oxidative stress markers, combined with a predominance of coronary MACE over peripheral events, is consistent with intimal atherosclerotic disease as a plausible biological interpretation in this cluster. The lowest vitamin D metabolite ratio in Cluster 3 may reflect inflammation-driven upregulation of vitamin D catabolism, further compounding the adverse metabolic milieu in this subgroup. Furthermore, the high proportion of hepatic steatosis comprises a link to metabolic inflammation, whereas the high rate of infections and lowest anti-HBs titers may indicate an additional disadvantageous immune regulation. The higher prevalence of central venous catheter vascular access in Cluster 3 may also have contributed to the increased burden of infection-related hospitalizations and sepsis mortality. However, central venous catheter access was not retained as an independent predictor in the multivariable survival analysis, suggesting that it is unlikely to fully explain the adverse outcomes observed in this cluster. Cluster 4 represented an intermediate phenotype of secondary hyperparathyroidism with moderate bone turnover, inflammation, and comorbidity burden and showed frequent reassignment over time. In line with our data, hyperparathyroid profiles in previous classification models also appeared to have more favorable outcomes.^15^

The addition of cluster membership as a binary predictor improved prediction of 1-year mortality beyond the clinical model and achieved predictive performance comparable with the individual cluster components. These findings suggest that cluster membership fully reflects the shared prognostic information captured by the individual biomarkers, providing an information-dense representation of biological risk. As such, the 4 cluster-derived phenotypes offer a clinically interpretable framework for risk stratification and understanding biological heterogeneity in the hemodialysis population.

Recent literature highlights the importance of systemic inflammation and oxidative stress as central drivers of vascular disease in CKD-MBD pathogenesis and outcomes.^23^^,^^24^ Our multidimensional clustering of established bone turnover markers with inflammatory stress signals achieved a clinical, phenotypic separation not attainable by single-analyte approaches. This was reflected in 2 low-event phenotypes with either true high bone turnover or an intermediary hyperparathyroid signature and 2 differential calcification phenotypes with osteoporotic fracture risk and detrimental MACE, inflammation, and mortality, respectively. As these biological signals are fundamental to CKD-MBD pathophysiology, the phenotypic structure is likely applicable across diverse hemodialysis populations. This integrated characterization represents a meaningful advancement over existing CKD-MBD classifications and provides a framework for personalizing therapeutic targets in accordance with Kidney Disease: Improving Global Outcomes’ consensus work explicitly advocating a move from single-analyte targets to integrated, personalized risk models of vascular and bone disease.^10^^,^^25^

The inflammatory profile and unfavorable outcomes of Cluster 3 are intriguing because systemic inflammation is not currently a treatment target in dialysis care. A recent randomized phase 2b trial of the IL-6–ligand inhibitor clazakizumab in maintenance hemodialysis showed a ∼90% reduction in hsCRP, favorable shifts in other inflammatory biomarkers, and an increase in serum albumin levels.^9^ A phase 3 study is ongoing to investigate clinical outcomes of anti-inflammatory use in hemodialysis (NCT05485961). The recent PISCES trial reported a striking reduction in cardiovascular mortality in patients receiving n-3 fatty acids.^26^ Because the trial was conducted in an unselected dialysis population, the mechanisms underlying this benefit remain unclear. A pathophysiology-driven intervention strategy, such as targeting anti-inflammatory or antioxidative therapies to patients in the inflammatory–oxidative Cluster 3, could be the subject of further study to help identify subgroups with concentrated risk and greater potential for therapeutic response.

Another key strength is that reclustering after 1 year reproduced the same subgroups and confirmed the high mortality of Cluster 3, showing that the approach can prospectively identify high-risk patients. Regarding cluster stability, transitions were not randomly distributed—Clusters 1, 2, and 3 showed limited crossflow between each other, with reassignment occurring predominantly into Cluster 4 in approximately 40% of patients from clusters 1 and 2, and 30% from Cluster 3. The highly directional nature of these transitions argues against analytical variability as an explanation. Instead, given that Cluster 4 carries more favorable outcomes and an intermediary biomarker profile, these shifts may reflect a treatment effect. This underscores the need for longitudinal phenotyping to distinguish stable biological phenotypes from dynamic, treatment-responsive states. Prospective studies in incident patients undergoing hemodialysis with repeated phenotyping are needed to confirm this interpretation.

This study has several limitations that should be acknowledged. First, although the clustering approach revealed biologically and prognostically distinct subgroups, external validation in independent hemodialysis cohorts is lacking. However, the rigorous sensitivity analysis in a multicenter cohort and the consistent results by reassessment of clusters after 1 year strengthen the internal validity and generalizability of our results. Second, although noxPTH® and iPTH were included as indicators of PTH bioactivity consistent with current guidance at the time of study design, the biomarker panel warrants critical appraisal considering updated consensus recommendations.^25^ BAP served as the primary confirmatory marker of bone turnover and aligns with currently recommended panels.^25^ Concerning renal clearance, all patients were on maintenance hemodialysis with comparable residual renal function, suggesting that relative differences between clusters still reflect true biological variation. TRAcP5b and intact procollagen type I N-propeptide represent preferred nonrenally cleared alternatives for future studies.^25^ The stable iPTH:noxPTH ratio across all clusters (range 1.5–1.6; *P* = 0.32) suggests results would be reproducible with conventional iPTH. Third, adjustment for dialysis vintage partly addresses potential survival–prevalence bias. Nonetheless, the association between biomarker profiles and outcomes requires confirmation in incident patients undergoing hemodialysis. In addition, calcification measures (*n* = 257) and repeat biomarker assessments (*n* = 148) were only accessible in a subset of the cohort, which may limit statistical power in longitudinal analyses. Fourth, skeletal characterization in this study was indirect, relying on biomarkers of bone turnover and fracture rates. The absence of bone mineral density assessment, bone turnover–specific imaging, or histomorphometric validation constrains the conclusions that can be drawn regarding skeletal biology and limits interpretation of fracture outcomes beyond association with cluster membership. These investigations should be incorporated in future studies aiming to validate the bone phenotypes identified here. Fifth, although cluster membership added prognostic value beyond individual biomarkers, the mechanistic underpinnings of these phenotypes require further investigation, ideally through integrative OMICs and interventional studies.

In conclusion, multidimensional biomarker clustering captures heterogeneity in the bone-vascular disease axis of CKD-MBD and identifies phenotypes exhibiting differential mortality risk and event patterns in patients undergoing maintenance hemodialysis. Inflammation and oxidative stress remain currently uncontrolled risk factors in association with adverse outcomes. Our results highlight the potential of pathophysiology-based patient classifications to address unmet needs in individualizing CKD-MBD management and advancing precision medicine in dialysis care.

## Disclosure

TBD and FPA are part of Immundiagnostik AG, Bensheim, Germany. KE, EZ, and DC received funding from ImmundiagnostikAG to perform the study. The other authors report no conflict of interest regarding this study.

## Supplementary Material

Supplementary File (pdf) **Figure S1.** Study flowchart. **Figure S2.** The correlation between iPTH and noxPTH for Clusters 1 to 4. **Figure S3.** DistribuPon of the cluster feature variables. Partitioning of participants into 4 clusters along 7 variables in the cohort after 1 year (*n* = 148). Relative differences from the mean (mean = 0) are visualized as z-scores for each cluster component. **Figure S4.** Sankey diagram showing the patient flow between clustering performed at baseline and after 1 year. **Table S1.** Test assay details. **Table S2.** Baseline characteristics of the whole cohort. **Table S3.** Patient distribution by center between clusters. **Table S4.** Leave-one-center-out (LOCO) clustering validation results. **Table S5.** One-year mortality from Kaplan-Meier analysis and adjusted cox-regression. **Table S6.** Incremental predictive value of cluster membership and biomarker components for 1-year mortality. **Table S7.** Dynamics of repeated clustering at 1 year in reference to the cluster-assignment at baseline.

## Footnotes

Supplementary File (PDF)
**Figure S1.** Study flowchart. **Figure S2.** The correlation between iPTH and noxPTH for Clusters 1 to 4. **Figure S3.** Distribution of the cluster feature variables. Partitioning of participants into 4 clusters along 7 variables in the cohort after 1 year (*n* = 148). Relative differences from the mean (mean = 0) are visualized as *z*-scores for each cluster component. **Figure S4.** Sankey diagram showing the patient flow between clustering performed at baseline and after 1 year. **Table S1.** Test assay details. **Table S2.** Baseline characteristics of the whole cohort. **Table S3.** Patient distribution by center between clusters. **Table S4.** Leave-one-center-out (LOCO) clustering validation results. **Table S5.** One-year mortality from Kaplan-Meier analysis and adjusted Cox-regression. **Table S6.** Incremental predictive value of cluster membership and biomarker components for 1-year mortality. **Table S7.** Dynamics of repeated clustering at 1 year in reference to the cluster-assignment at baseline.
