# Supplementary material for: Data-Driven Clustering of Secretory Bone and Inflammatory Markers to Subphenotype Patients Undergoing Hemodialysis
Source: Kidney Int Rep. 2026 Aug 14;11(11):107019. doi: 10.1016/j.ekir.2026.107019 (PMC13634881; doi:10.1016/j.ekir.2026.107019)
Supplement: Supplementary file 1 [file mmc1.pdf]

Supplemental Material

Data-driven clustering of secretory bone and inflammatory markers to sub-phenotype hemodialysis patients

Simon Aberger, Ameen A. Hanna, Vianda Stel, Nick Chesnaye, Emanuel Zitt, Daniel Cejka, Thomas B. Dschietzig, F. P. Armbruster, Hildegard Hafner-Gießauf, Maximilian Waller, Corinna Schabhüttl, Alexander H. Kirsch, Alexander R. Rosenkranz, Kathrin Eller

R Packages for the clustering solution:

- “DescTools” (Winsorize)
- “cluster” (Gower, PAM)
- “factoextra” (visualization and silhouette width)
- “fpc” (cluster stability)

Selection of cluster components:

Variable selection for clustering was guided by pathophysiological considerations, focusing on biological processes relevant to bone disease, mineral metabolism and vascular pathology in hemodialysis patients. A prespecified set of biomarkers, encompassing markers of osteoblast/osteoclast input (noxPTH, OPG/sRANKL), bone resorption (betaCL), oxidative stress (PerOx®), antioxydative capacity (ImAnOx®) and systemic inflammation (hsCRP) with high discriminatory potential was used. Moderate correlations between marker pairs were considered acceptable for $r < 0.5$, reflecting the biological interdependence of bone turnover and oxidative stress pathways. Prior to clustering, we verified the preservation of multidimensional structure using principal component analysis, confirming that no single marker disproportionately influenced the distance matrix. Additional markers such as iPTH, bone-specific AP, MPO and sclerostin were subsequently analyzed descriptively across clusters to avoid redundancy and colinearity.

Cluster analysis rationale:

Gower distance was implemented as a background tool for its well-documented advantages in clustering clinical datasets, particularly when variables differ in scale or type. Our dataset

comprised exclusively numerical clinical biomarkers measured on heterogeneous scales, which makes the calculated distances equal to range normalized Manhattan distance. The implementation of Gower distance as a background tool would allow inclusion of mixed data types in future projects.

The use of the Partitioning Around Medoids (PAM) algorithm further strengthened our clustering approach for clinical biomarker data. Unlike k-means clustering, which relies on the mean as the cluster center, PAM identifies medoids (actual observations within the dataset) as cluster representatives. This enhances robustness to outliers and strengthens clinical interpretability, as each cluster is anchored to a real patient profile. Moreover, PAM assigns individuals definitively to a single cluster, supporting discrete subgroup identification. Our dataset, comprising 471 patients and fewer than 10 biomarkers, is well-suited to PAM, which does not require high-dimensional data or prior dimensionality reduction. Its compatibility with the Gower distance metric further enables flexible scaling and integration of heterogeneous clinical variables. Together, these characteristics make PAM a well-matched and interpretable clustering algorithm for patient-level clinical data.

Subdistribution Hazard Model:

We fitted a sequence of nested Fine–Gray subdistribution hazard models using the cmprsk package in R. The event of interest was death within 365 days, with kidney transplantation modeled as a competing event ($n = 16$). Variables were introduced hierarchically in biologically meaningful blocks. Model fit was evaluated using the Akaike Information Criterion (AIC), calculated from the model log-likelihood and number of estimated parameters ($AIC = -2 \times \log L + 2 \times k$). The procedure retains the variable if the improvement in model fit (the reduction in -2 log(Likelihood)) is greater than the penalty for adding it (2 points). Lower AIC values indicate improved balance between fit and complexity; $\Delta AIC > 2$ was considered evidence of a meaningful improvement. Akaike weights were used to compare the relative likelihood of each model being the best among candidates. All continuous predictors were entered untransformed after checking linearity.

Sensitivity analysis:

To evaluate the robustness of the clustering results across centers, we performed a leave-one-center-out (LOCO) analysis. In each iteration, data from one study center were excluded and clustering was repeated on the remaining centers. The resulting cluster solution was compared to the full dataset clustering using the Adjusted Rand Index (ARI) to assess stability of the cluster assignments (*ARI train vs full*). In addition, individuals from the excluded center were assigned to clusters based on the 3-center solution and their agreement with the full dataset clustering was evaluated (*ARI test vs full*). This procedure allowed us to assess both the stability of the clustering structure when one center was omitted and the generalizability of the cluster assignments to previously unseen data.

In addition to the leave-one-center-out (LOCO) procedure, we performed a longitudinal validation of the clustering algorithm in the subset of patients (n = 148) who had repeated biomarker measurements after one year. Clustering was repeated on the follow-up dataset, and the resulting cluster assignments were compared to the baseline clusters. To visualize individual transitions between clusters, we generated a Sankey diagram. To evaluate prognostic relevance, we extended follow-up in this subgroup to record mortality events and compared cumulative survival across baseline clusters using Kaplan–Meier curves with the log-rank test.

Supplemental Tables:

Supplemental Table 1: Test assay details.

Parameter	Method	Manufacturer	Catalog No.	Antibodies / Assay Principle
**iPTH**	ECLIA	Roche	8928380190	Sandwich assay principle: biotinylated monoclonal antibody reacts with the N-terminal fragment (1-37) and a monoclonal antibody labeled with a ruthenium complex reacts with the C-terminal fragment (38-84).
**nox-PTH**	ECLIA	Roche (IDK sample preparation method)	8928380190	The same antibodies as used in the iPTH assay were applied, with a monoclonal rat anti-human oxPTH antibody from IDK used for sample pretreatment. Nox-PTH is separated from bound ox-PTH and measured in the iPTH ECLIA test.
**beta-Crosslaps**	ECLIA	Roche	9005773190	Sandwich assay principle: two monoclonal antibodies, each recognizing linear β-8AA octapeptides (EKAHD-β-GGR). The first antibody is biotinylated, and the second is labeled with a ruthenium complex
**OPG**	ELISA	IDK	K 1017	Sandwich assay principle: microplate coated with monoclonal mouse-anti-OPG-antibody. Biotinylated polyclonal anti-OPG-antibody and streptavidin-peroxidase-conjugate is added and a “sandwich” of 1st antibody – OPG - biotinylated antibody – streptavidin-peroxidase-conjugate is formed.
**ImAnOx**	photometric assay	IDK	KC5200	The determination of the antioxidative capacity is performed by the reaction of antioxidants in the sample with a defined amount of exogenously provided hydrogen peroxide (H2O2) The antioxidants in the sample eliminate a certain amount of the provided hydrogen peroxide. The residual H2O2 is determined photometrically by an enzymatic reaction which involves the conversion of TMB to a colored product.
**PerOx**	colorimetric assay	IDK	KC5100	The determination of the peroxides is performed by the reaction of a peroxidase with peroxides in the sample followed by the conversion of TMB to a colored product.
**total sRANKL**	ELISA	IDK	K 1016	Sandwich assay principle: microplate coated with a high-affinity polyclonal anti-human OPG antibody.Biotinylated monoclonal anti-human sRANKL antibody is added. A streptavidin horseradish-peroxidase conjugate is added, which specifically binds to biotin.
**MPO**	ELISA	IDK	K 6631B	Sandwich assay principle: microplate coated with a high-affinity polyclonal anti-human MPO antibody. a peroxidase-conjugated polyclonal anti-human MPO antibody is added and a “sandwich” of capture antibody – human MPO – Peroxidase conjugate is formed.
**Sclerostin**	ELISA	Biomedica	BI-20492	Sandwich assay principle: microtitre plate coated with polyclonal goat anti human Sclerostin antibody. The second Antibody is a monoclonal mouse anti human Sclerostin antibody - biotin labeled. Detection is performed by the addition of streptavidin conjugated to HRPO.

Supplemental Table 2: Baseline characteristics of the whole cohort.

Biological parameters	Overall (N=471)
Age [a]	71 (61–78)
Male sex (%)	308 (66%)
BMI	25.3 (22.0–29.1)
**Dialysis parameters**	**Overall (N=471)**
Dialysis vintage [mo]	29 (12–54)
HD [h/week]	11.60 (1.11)
Blood flow [mL/min]	274 (36)
RKF [mL/day]	659 (548)
ktV (single pool)	1.23 (0.26)
AV Fistula (%)	249 (54%)
**CVD burden**	**Overall (N=471)**
Smoking status (%)	92 (28%)
CHF (%)	115 (25%)
COPD (%)	56 (12%)
CAD (%)	180 (38%)
PAD (%)	142 (30%)
Carotid disease (%)	100 (21%)
AHT (%)	444 (94%)
Diabetes (%)	201 (43%)
Hepatic steatosis (%)	67 (14%)
Aortic calcification grade	1.86 (0.93)
Valve calcification (%)	87 (40%)
**Medication**	**Overall (N=471)**
Calcimimetics (%)	161 (34%)
Active VD (%)	276 (59%)
Phosphate binders (%)	346 (73%)
Antiresorptives (%)	12 (2.5%)
ESA (%)	315 (67%)
IV Iron (%)	222 (47%)
**Laboratory paramters**	**Overall (N=471)**
iPTH [pg/mL]	268 (164–433)
noxPTH® [pg/mL]	173 (102–284)
iPTH:noxPTH ratio	1.64 (1.54)
Beta-CL [ng/mL]	1.57 (1.10–2.33)
OPG [pmoL/mL]	23 (16–32)
ImAnOx® [µmoL/mL]	308 (282–339)
PerOx® [µmoL/mL]	290 (155–466)
sRANKL [ng/mL]	15 (6–98)
hsCRP [mg/L]	6 (3–14)
MPO [IU/mL]	297 (220–371)
1,25-D3 [ng/mL]	11 (6–19)
24,25-D3 [ng/mL]	0.30 (0.20–0.50)
25-D3 [ng/mL]	28 (14–44)
VMR (25/24.25D3)	78 (36)
BAP [ng/mL]	20 (14–28)
Sclerostin [pmoL/L]	107 (77–151)
Phosphate [mmoL/L]	1.78 (1.47–2.19)
Calcium [mmoL/L]	2.13 (2.00–2.23)
Albumin g/dL[]	4.00 (3.67–4.30)
ALP [U/L]	88 (69–117)
Hemoglobin [g/dL]	11.20 (10.40–12.00)
Ferritin [µg/L]	510 (302–798)

TSAT (%)	23 (17–31)
Cholesterol [mg/dL]	149 (122–185)
LDL [mg/dL]	77 (55–107)
HDL [mg/dL]	42 (35–50)
Anti-HBs [IU/mL]	35 (2–183)

Footnote: *Values are presented as median (Q1–Q3) for age, dialysis vintage, BMI, and laboratory parameters; mean (SD) for haemodialysis hours/week, blood flow, residual kidney function, Kt/V, PTH/noxPTH ratio, vitamin D metabolite ratio, and abdominal aortic calcification grade; and n (%) for categorical variables.*

Supplemental Table 3: Patient distribution by center between clusters.

	**Center**			
**Cluster**	**1**	**2**	**3**	**4**
**1**	20 (26.7%)	22 (29.3%)	18 (24.0%)	15 (20.0%)
**2**	36 (30.8%)	35 (29.9%)	20 (17.1%)	26 (22.2%)
**3**	38 (27.5%)	43 (31.2%)	27 (19.6%)	30 (21.7%)
**4**	34 (24.1%)	45 (31.9%)	34 (24.1%)	28 (19.9%)

Supplemental Table 4: Leave-one-center-out (LOCO) clustering validation results.

**Center left out**	**n_train**	**n_test**	**ARI train vs full (No Std)**	**ARI test vs full (No Std)**	**ARI train vs full (Std)**	**ARI test vs full (Std)**
1	343	128	0.349	0.424	0.844	0.885
2	326	145	0.491	0.301	0.854	0.868
3	372	99	0.956	0.86	0.767	0.69
4	372	99	0.349	0.352	0.854	0.888

Footnote: *For each run, one center was excluded and clustering was performed on the remaining three centers. The resulting cluster assignments were compared to the full dataset clustering using the Adjusted Rand Index (ARI). ARI train vs full reflects whether the clustering solution obtained with three centers is consistent with the clustering from all four centers (i.e., does leaving one center out change the clustering?). ARI test vs full evaluates whether the left-out center, when assigned to clusters based on the 3-center solution, agrees with the full dataset clustering (i.e., generalizability to unseen data). Results show that without center-based standardisation, clustering solutions vary substantially across centers, whereas with standardisation, both the stability of the clustering (train vs full) and its generalizability (test vs full) are much improved. ARI: Adjusted Rand Index. Std: center-based standardisation.*

Supplemental Table 5: One-year mortality from Kaplan-Meier analysis and adjusted cox-regression.

Cluster	1-year mortality n/N (%)	aHR (95% CI)	p-value
1	4/75 (5.6%)	Ref.	Ref.
2	17/117 (14.7%)	2.70 (0.9-8.0)	0.07
3	51/138 (37.1%)	7.93 (2.8-21.9)	<0.01
4	14/141 (10.0%)	1.77 (0.5-5.3)	0.31

Footnote: *One-year mortality was estimated from Kaplan–Meier survival curves with kidney transplantation treated as a censoring event. Hazard ratios (HR) and 95% confidence intervals (CI) were derived from a Fine–Gray model with Cluster 1 as the reference group and adjusted for baseline confounders (age, dialysis vintage, and albumin). Reported p-values correspond to Wald tests.*

Supplemental Table 6: Incremental predictive value of cluster membership and biomarker components for 1-year mortality.

Cluster	IDI	NRI	p-value
M2 vs M1	0.07	0.79	<0.05
M3 vs M1	0.06	0.70	<0.05
M4 vs M2	0.01	0.17	0.75
M4 vs M3	0.01	0.21	0.37

Footnote: *Integrated Discrimination Improvement (IDI), Net Reclassification Improvement (NRI).*

Supplemental Table 7: Dynamics of repeated clustering at 1 year in reference to the cluster-assignment at baseline.

Cluster at baseline	Percent stayed	Percent changed
1	59.1	40.9
2	58.4	41.6
3	68.3	31.7
4	35.2	64.8

Supplemental Figures:

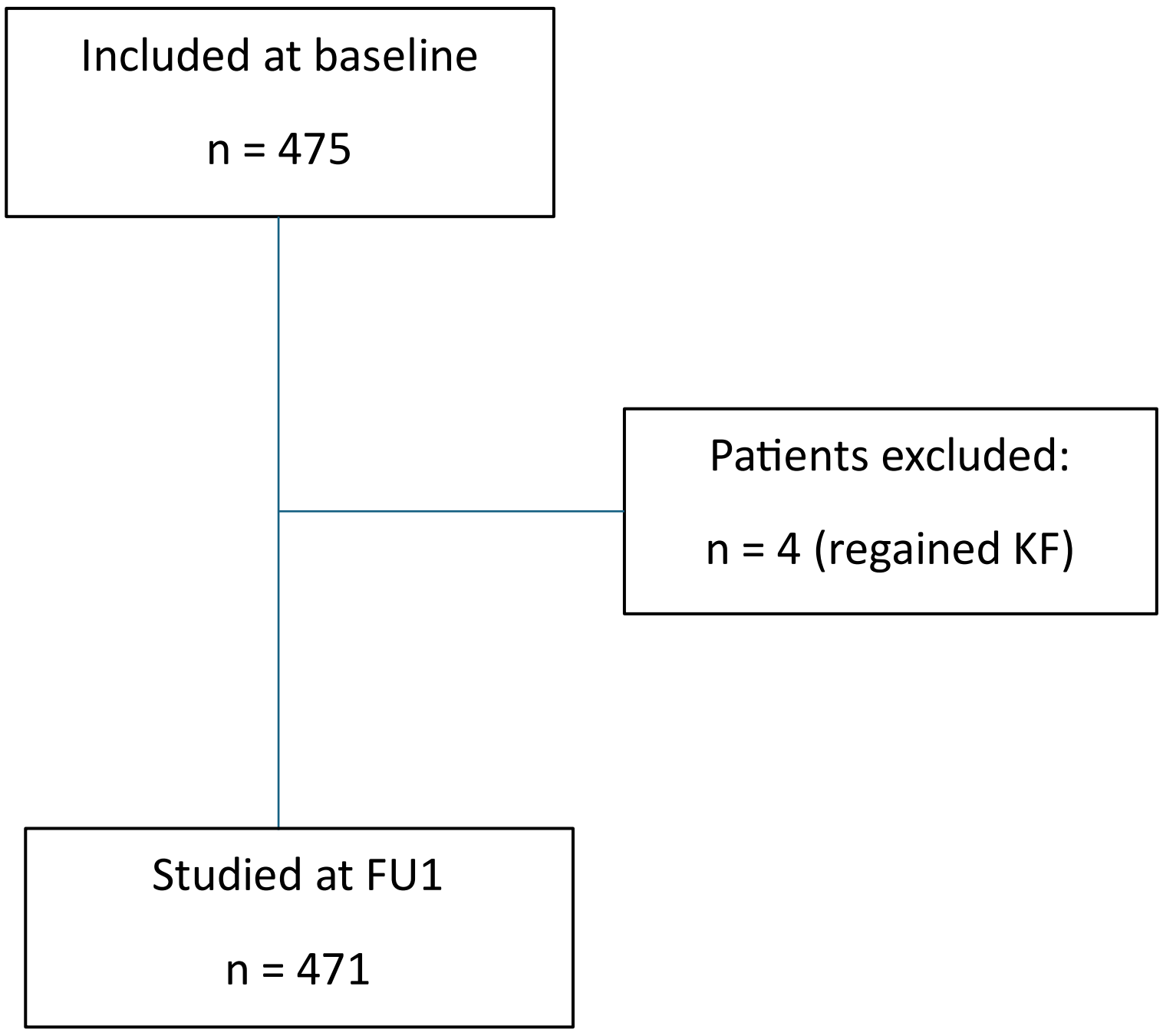

Supplemental Figure 1. Study flowchart

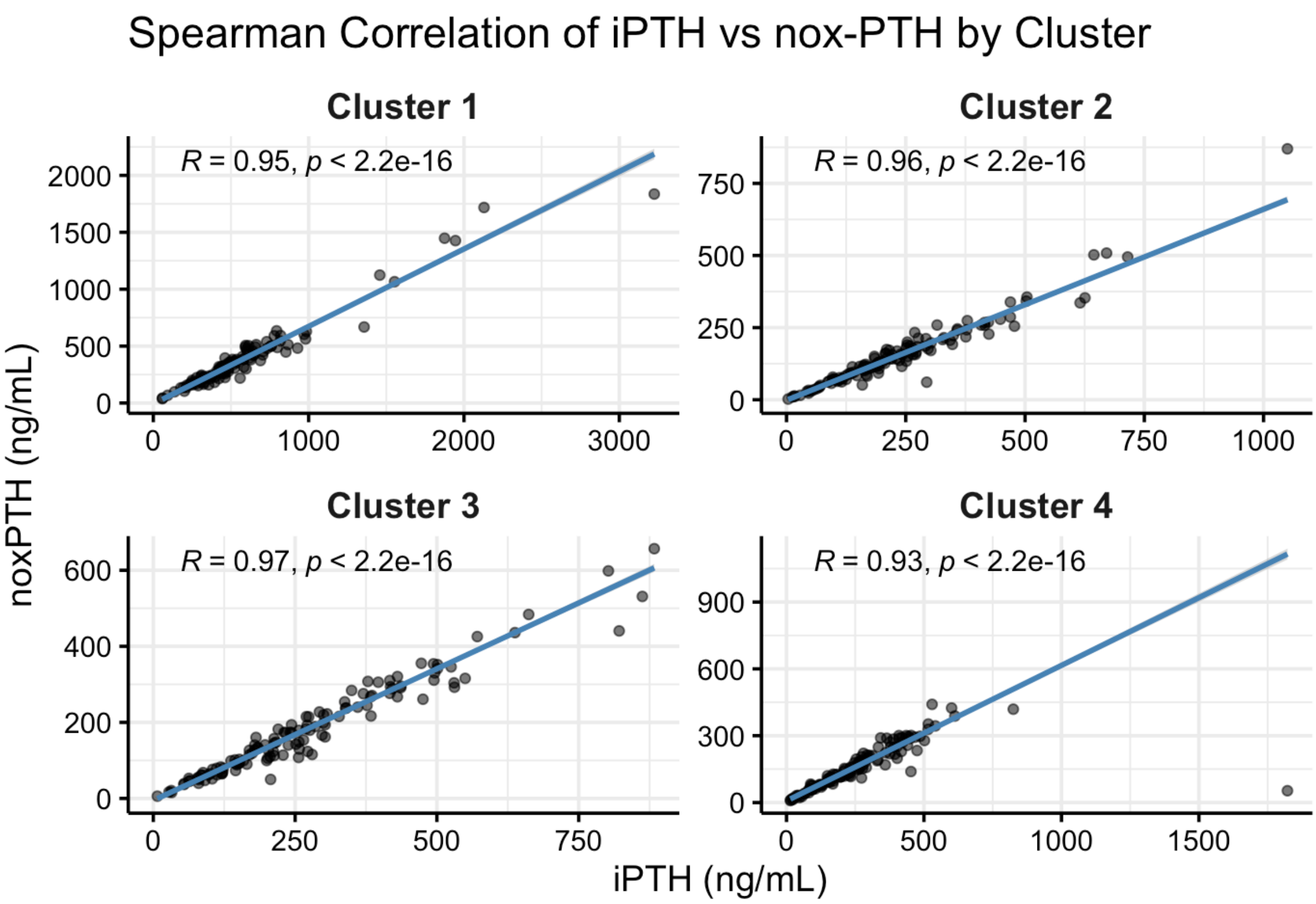

Supplemental Figure 2. The correlation between iPTH and noxPTH for Clusters 1 to 4. Spearman correlation coefficient and p-value are shown.

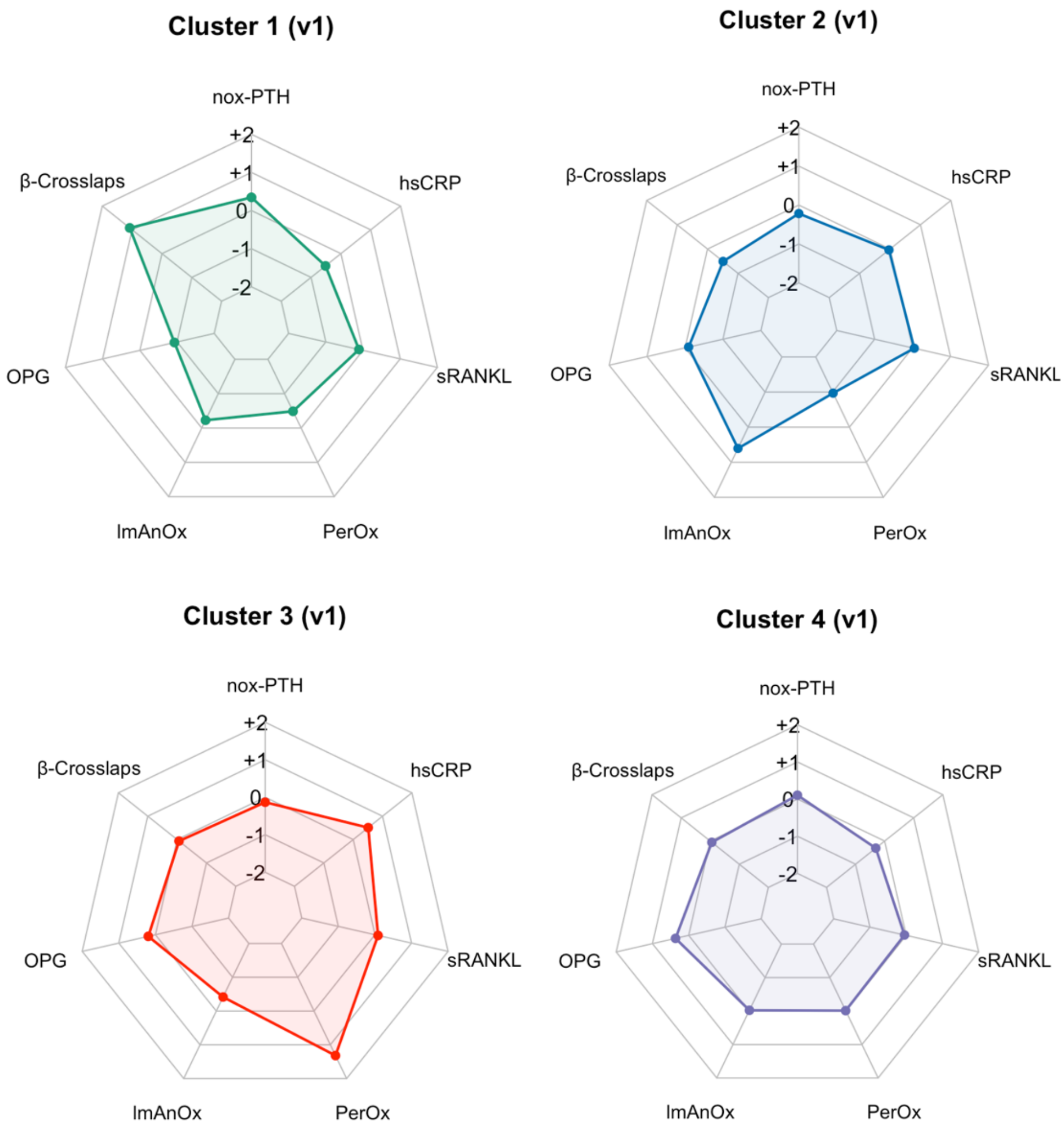

Supplemental Figure 3. Distribution of the cluster feature variables. Partitioning of participants into 4 clusters along 7 variables in the cohort after 1 year (n = 148). Relative differences from the mean (mean = 0) are visualized as z-scores for each cluster component.

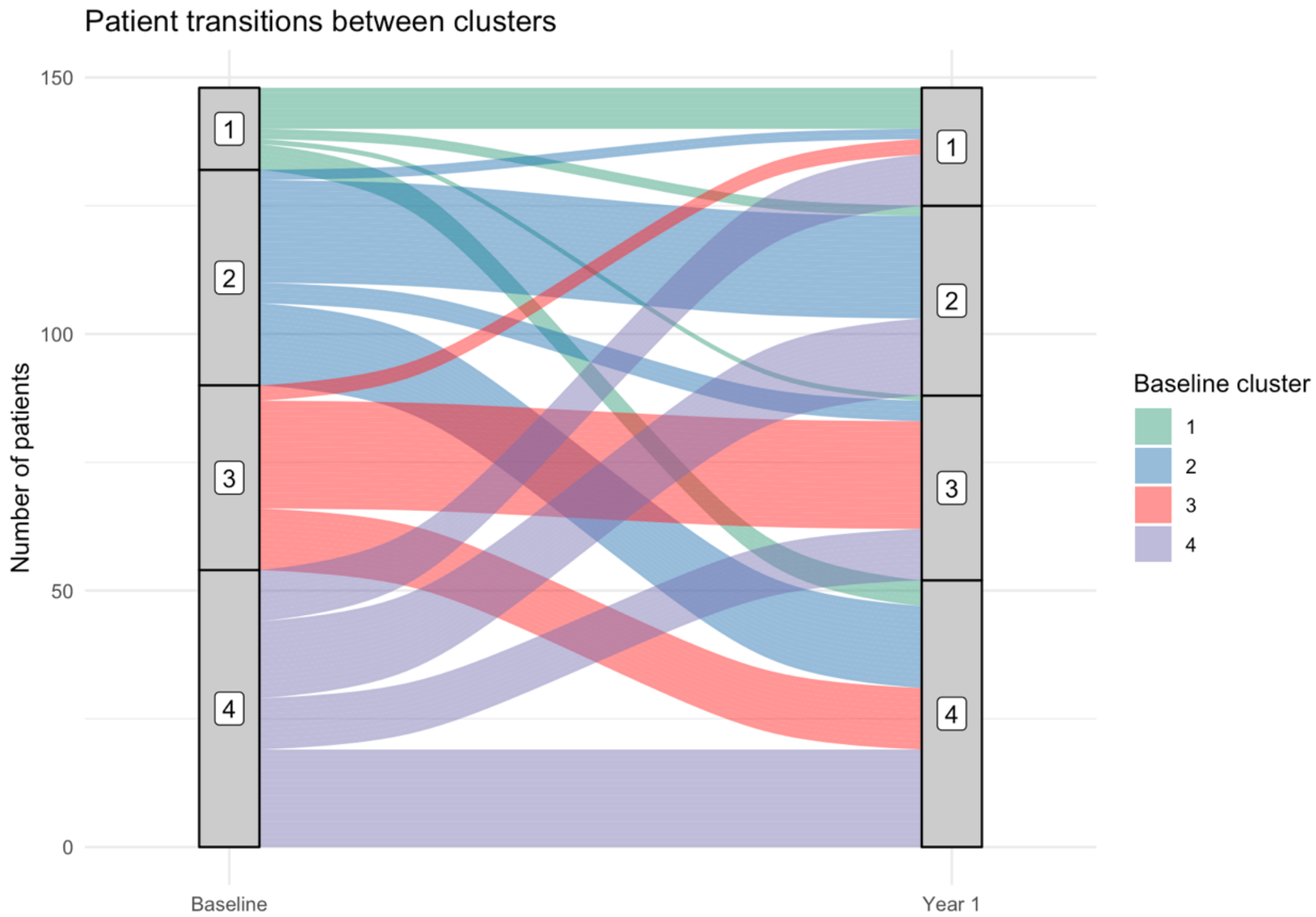

Supplemental Figure 4. Sankey diagram showing the patient flow between clustering performed at baseline and after 1 year.
